# Guideline of guidelines: Peyronie's disease

**DOI:** 10.1111/bju.70201

**Published:** 2026-03-08

**Authors:** Francesco Chierigo, Giuseppe Fallara, Marco Tozzi, Andrea Salonia, Matteo Ferro, Hussain M Alnajjar, Asif Muneer, Karl H. Pang

**Affiliations:** ^1^ Department of Urology, Department of Health Science, ASST Santi Paolo e Carlo University of Milan Milan Italy; ^2^ Vita‐Salute San Raffaele University Milan Italy; ^3^ Division of Experimental Oncology/Unit of Urology URI, IRCCS Ospedale San Raffaele Milan Italy; ^4^ Division of Surgery and Interventional Science University College London London UK; ^5^ Department of Urology University College London Hospitals NHS Trust London UK; ^6^ National Institute for Health and Research, Biomedical Research Centre University College London Hospitals London UK; ^7^ Department of Urology Chelsea and Westminster Hospital NHS Foundation Trust London UK

**Keywords:** Peyronie's disease, penile curvature, tunical plication, tunical incision and grafting, penile prosthesis, erectile dysfunction, surgical management, non‐surgical treatment, intralesional therapy, sexual function

## Abstract

**Objective:**

To compare major Peyronie's disease (PD) guidelines, highlight key similarities and differences among panel recommendations, and identify areas requiring further research.

**Methods:**

An extensive review was conducted to analyse and compare diagnostic and treatment recommendations from publicly available guidelines published by the American Urological Association, European Association of Urology, Canadian Urological Association, and the International Society of Sexual Medicine. Key similarities and differences regarding PD definition, evaluation, non‐surgical treatments, and surgical management were systematically compared.

**Results:**

Areas of general consensus across guidelines include the importance of comprehensive history‐taking for PD diagnosis and the role of intracavernosal injection as the ‘gold standard’ for assessing penile deformity prior to invasive intervention. Shared decision‐making and thorough patient counselling are universally emphasised. Plication or incision and grafting surgery is generally reserved for patients with preserved erectile function, whereas penile prosthesis implantation is the preferred surgical option for those with erectile dysfunction unresponsive to medical therapy. Non‐surgical treatments remain an area of controversy due to limited evidence of efficacy; however, intralesional injections are recognised by all panels as a potential treatment option, especially in the acute phase. Further research into PD pathophysiology and rigorous outcomes studies are needed to inform novel treatments and refine surgical management strategies.

**Conclusion:**

While major urological societies demonstrate substantial consensus on several aspects of PD evaluation and management, key areas of divergence remain, underscoring the need for further research to guide evidence‐based care.

**Abbreviations**
BoNT‐A: botulinum neurotoxin‐A
CCH: collagenase clostridium histolyticum
CUA: Canadian Urological Association
EAU: European Association of Urology
ED: erectile dysfunction
ESSM: European Society of Sexual Medicine
ESWT: extracorporeal shockwave therapy
ICI: intracavernosal injection
IIEF: International Index of Erectile Function
ISSM: International Society for Sexual Medicine
PD: Peyronie's disease
PDDU: penile duplex Doppler ultrasonography
PDE5i: phosphodiesterase type 5 inhibitors
PDQ: Peyronie's Disease Questionnaire
PTT: penile traction therapy
VED: vacuum erection device

## Introduction

Peyronie's disease (PD) is a chronic fibrotic disorder of the tunica albuginea that causes penile curvature, pain, and often erectile dysfunction (ED), impairing sexual function [1]. The condition evolves from an acute, symptomatic phase—with progressive deformity and pain—to a chronic, stable phase after 6–12 months. Beyond physical changes, PD markedly affects psychological and relational well‐being, frequently leading to shame, reduced self‐confidence, and partner sexual dysfunction [2, 3, 4, 5, 6, 7, 8, 9, 10, 11].

Its reported prevalence ranges from 0.4% to 9% in the general population and up to 15–20% in men with diabetes or prostate cancer. Established risk factors include prior penile trauma, hypertension, older age, and smoking [10, 12, 13, 14, 15]. Discrepancies across studies reflect variable definitions, under‐recognition, and patients’ reluctance to seek care [16, 17, 18].

Management ranges from conservative to surgical, aiming to relieve pain, correct curvature, stabilise plaque, preserve penile length, and restore penetrative capacity [1, 19]. However, most studies are small, methodologically limited, and use non‐standardised outcomes, while the fibrotic mechanisms remain incompletely understood [20, 21, 22]. Variability in clinician expertise and access to care further contributes to suboptimal management [23, 24].

To address these challenges, major professional societies—including the American Urological Association, International Society for Sexual Medicine (ISSM), Canadian Urological Association (CUA), and European Association of Urology (EAU)—have issued guidelines to standardise diagnosis and treatment [1, 25, 26, 27]. Yet, many recommendations rely on expert opinion, and differences among guidelines persist. This review compares current recommendations, outlining areas of consensus, disagreement, and gaps requiring future research.

## Methods

### Guidelines Identification and Methodology

We systematically searched PubMed, the Excerpta Medica dataBASE (EMBASE), Scopus, Google Scholar, and the Cochrane Library for guidelines on PD up to 30 June 2025, using the terms ‘Peyronie,s Disease’, ‘Penile Recurvatum’, and ‘Induratio penis plastica’ with ‘review,’ ‘systematic review,’ and ‘meta‐analysis’ filters in PubMed. Only English‐language guidelines were included. Manual searches of international and national society websites and reference lists supplemented the database search.

Documents not meeting a clinical guidelines framework or lacking comprehensive patient management strategies (e.g., focusing solely on imaging) were excluded. Four major guidelines were selected for comparison—AUA, ISSM, CUA, and EAU [1, 25, 26, 27]. The Asia‐Pacific Society of Sexual Medicine (APSSM) consensus statement on penile reconstructive and prosthetic surgery was excluded as it does not extensively cover PD [28]. Similarly, national guidelines largely derived from European (EAU, European Society of Sexual Medicine [ESSM]), American (AUA, ISSM), or Canadian (CUA) recommendations—such as those issued by the French Urological Association (AFU) [29] ‐ were not included in the present synthesis because of their substantial overlap with existing international guidelines. Development processes for the included guidelines are detailed in the Data S1.

### Review and Comparative Summary of Guidelines Content

Two authors (F.C. and G.F.) independently reviewed all four guidelines, pre‐defining key domains—diagnosis, non‐surgical management, and surgical treatment. Data were abstracted into structured tables (Tables 1, 2, 3). Where explicit recommendations were lacking, relevant panel positions were inferred from contextual discussion. Recommendations were classified as ‘recommended’, ‘may consider’, ‘unclear benefits’, or ‘recommended against’, with unaddressed topics marked ‘not addressed’. This comparative analysis outlines current consensus, practice variation, and evidence gaps in PD management.

**Table 1:** Peyronie's disease evaluation.

	AUA (2015)	ISSM (2016)	CUA (2018)	EAU (2025)
Definition of ‘stable’ phase PD	Symptom duration ≥12 months; stable PD symptoms for 3–6 months (Clinical principle)	Symptom duration >6–12 months; stable PD symptoms for >3–6 months (Expert opinion)	Symptom duration >6–12 months; stable PD symptoms for 3–6 months	Symptom duration >9–12 months; stable PD symptoms for 3–6 months (Level 2b; Strong)
History and physical examination	Recommended (Clinical principle)	Recommended (Clinical principle)	Recommended (Level 4; Grade C)	Recommended (Strong)
Questionnaires	Not addressed	RecommendedPDQ^b^(Level 4; Grade B)	May consider IIEF^a^ PDQ^b^ *(*Level 3; Grade C)	May consider IIEF^a^ PDQ^b^ (Weak)
Mental health referral	May consider	May consider	May consider	Not addressed
Home photograph (full erection)	May consider	May consider Could be sufficient for baseline and monitoring if no invasive treatment is undertaken	May consider	May considerShould not replace curve assessment with ICI
ICI	Recommended prior to invasive intervention (Expert opinion)	Recommended prior to invasive intervention (Expert opinion)	Recommended (Level 4; Grade C)	Recommended (Level 4; Weak)
Doppler ultrasonography	May consider(Expert opinion)	May consider(Level 3; Grade C)	May consider(Level 4; Grade C)	May consider Assessment of penile haemodynamics and vascular anatomy (Level 2a; Weak) NOT recommended for plaque size estimation (Level 3; Weak)
Biothesiometry	May consider	May consider	Not addressed	Not addressed
MRI	Not addressed	Recommended against (Level 3; Grade C)	Recommended against	Recommended against
CT	Not addressed	Recommended against (Level 3; Grade C)	Recommended against	Recommended against
X‐ray	Not addressed	Recommended against (Level 3; Grade C)	Recommended against	Not addressed

Green = recommended, yellow = may consider, red = recommendation against, grey = not addressed.

^a^ International Index of Erectile Function.

^b^ Peyronie’s Disease Questionnaire.

**Table 2:** Non‐surgical treatment options for PD.

Treatment	AUA (2015)	ISSM (2016)	CUA (2018)	EAU (2025)
NSAIDs	Recommended For pain in active phase (Expert opinion)	Not addressed	Recommended For pain in active phase	May consider For active‐phase pain (Level 4; Strong)
Vitamin E	Recommended against (Grade B)	Recommended against (Grade B; Level 2)	Recommended against	Recommended against (Grade 3c; Strong)
Tamoxifen	Recommended against(Grade C)	Recommended against*(*Grade B; Level 2)	Recommended against	Recommended against (Grade 3c; Strong)
Procarbazine	Recommended against(Grade C)	Not addressed	Recommended against	Recommended against (Grade 3c; Strong)
Omega‐3 fatty acids	Recommended against(Grade B)	Not addressed	Not addressed	Recommended against (Grade 3c; Strong)
Vitamin E + L‐carnitine	Recommended against(Grade B)	Recommended against(Grade B; Level 2)	Recommended against	Recommended against (Grade 3c; Strong)
Potassium para‐aminobenzoate	Unclear benefits (no recommendation)	Recommended against	May consider(Level 3; Grade C)	Recommended against (Grade 3c; Strong)
Colchicine	Unclear benefits (no recommendation)	Recommended against(Grade B; Level 2)	May consider(Level 3; Grade C)	Recommended against (Strong)
Pentoxifylline	Unclear benefits (no recommendation)	Recommended against(Grade B; Level 2)	May consider(Level 3; Grade C)	Recommended against (Grade 3c; Strong)
Co‐enzyme Q10	Unclear benefits (no recommendation)	Not addressed	May consider(Level 3; Grade C)	Not addressed
PDE5i	Not addressed	Recommended against To benefit penile deformity with PD ED treatment not addressed (Grade B; Level 2)	May consider	May consider To treat concomitant ED To optimise penetration if deformity makes penetration intercourse difficult (Weak)
Electromotive therapy + topical verapamil	Recommended against(Grade C)	Recommended against(Level 3; Grade B)	Recommended against	Recommended against (Level 3c)
Topical therapy (monotherapy)	Unclear benefits (no recommendation)	Not addressed	Unclear benefits with topical verapamil (no recommendation)(Level 4; Grade C)	Recommended against (Level 3c)
PTT	Unclear benefits (no recommendation)	May consider (Level 3; Grade C)	May consider (Level 4; Grade C)	May consider (Level 3c; Weak)
ESWT	May consider For pain only Do not offer for plaque size or penile curvature (Grade B)	May consider For penile pain Panel experts suggest potential benefit on symptom stabilisation (does NOT correct deformity) (Level 3; Grade B)	May consider For pain only Do not offer for plaque size or penile curvature (Level 2, Grade C)	May consider For pain only (Level 2b; Weak) Do not offer for plaque size or penile curvature (Level 2b, Strong)
Radiation therapy	Recommended against (Grade C)	Not addressed	Recommended against	Not addressed
CCH	May consider (Grade B)	May consider (Level 2; Grade B)	May consider (Level 2; Grade B)	Recommended (Level 1b; Strong)
Inclusion criteria (optimal patient characteristics)	30–90° curve ‘Stable’ symptoms Intact erectile function with or without medical therapy	30–90° curve No isolated hourglass Non‐calcified plaque Plaque NOT located at penile base ‘Stable’ symptoms Intact erectile function with or without medical therapy	30–90° curve No isolated hourglass Non‐calcified plaque ‘Stable’ symptoms Intact erectile function with or without medical therapy	>30° curve Dorsal or later curve Patients who desire non‐surgical treatment ‘Stable’ phase
IFN‐α2b	May consider (Grade C)	May consider (Level 2; Grade B)	May consider (Level 2; Grade B)	May consider (Level 2b; Weak)
Optimal patient characteristics	>30° curve ‘Stable’ symptoms Non‐calcified plaque	Not addressed	>30° curve Non‐calcified plaque	>30° curve Dorsal or lateral curve Patients who desire non‐surgical treatment ‘Stable’ phase
Verapamil/nicardipine	May consider (Grade C)	May consider (Level 3; Grade C)	May consider (Level 2; Grade B)	Recommended against *(*Level 4)
Optimal patient characteristics	Not addressed	Not addressed	Not addressed	Not addressed
Corticosteroids	Not addressed	Recommended against	Recommended against (Grade C)	Recommended against (Level 3c; Strong)
Hyaluronic acid	Not addressed	Not addressed	Unclear benefits (no recommendation)	Recommended against (Level 3c; Strong)**Exception: inside clinical trials**
BoNT‐A	Not addressed	Not addressed	Unclear benefits (no recommendation)	Recommended against (Level 3c)
PRP	Not addressed	Not addressed	Not addressed	Recommended against (Level 3c; Strong)**Exception: inside clinical trials**
Combined therapies	Unclear benefits (no recommendation)	May consider	Not addressed	May consider (Level 3c; Weak)

Green = recommended, yellow = may consider, blue = unclear benefits, red = recommendation against, grey = not addressed.

**Table 3:** Criteria for surgical management options of stable PD.

	AUA (2015)	ISSM (2016)	CUA (2018)	EAU (2025)
Plication	Stable PD Adequate erectile rigidity with or without pharmacotherapy or VED (Grade C)	Stable PD Adequate erections with or without pharmacotherapy Adequate penile length Minimal to moderate curvature Absence of hourglass causing hinge (Level 3; Grade B)	Stable PD Adequate erections with or without pharmacotherapy Adequate penile length Curve that is ‘reasonably correctable with this approach’ Minimal/absent hourglass + hinge (Level 3; Grade C)	Stable PD for at least 3 months (after 12 months for the onset of symptoms) (Level 2b; Strong) Functional impairment (Level 2b; Strong) Adequate erections with or without pharmacotherapy Adequate penile length Absence of complex deformity (hourglass, hinge) ‘Non‐severe’ curvature (Level 3; Weak)
Preferred plication technique	Not specifically addressed	No surgical procedure with proven superiority	Dependent on surgeon and patient factors	Dependent on surgeon and patient preference No procedure with proven superiority
PIG or PEG	Stable PD Adequate erectile rigidity with or without pharmacotherapy or VED (Grade C)	Stable PD ‘Good’ erectile function Significant curvature and/or Indentation/hourglass deformity (with or without) Concerns regarding further penile length loss (Level 3; Grade B)	Stable PD Adequate erections with or without pharmacotherapy Short penile length Severe curvature >60° Large penile plaques Complex indentation or hourglass (Level 3; Grade C)	Stable PD Adequate erections with or without pharmacotherapy Significant penile shortening Severe penile curvature (>60°commonly cited but, per the Guidelines panel experts there is no unanimous consensus to support this threshold) Complex deformities (hourglass, hinge) (Level 3; Weak)
Preferred graft material	Not addressed	No ideal/preferred graft Synthetic grafts not recommended	No preferred graft Recommend against synthetic grafts (Level 3; Grade C)	No preferred graft material Recommend against synthetic grafts (Strong)
Penile prosthesis with adjunctive procedures*	Stable PD ED and/or penile deformity that impairs coitus (despite pharmacotherapy or VED) (Grade C)	Stable PD ED with inadequate response to pharmacotherapy or VED Complex deformity (Level 3; Grade B)	Stable PD ED non‐responsive to pharmacotherapy Severe deformity refractory to non‐surgical management Failed plication or grafting Profound penile instability (buckling or hinge) (Level 3; Grade C)	Stable PD for at least 3 months (after 12 months for the onset of symptoms) (Level 2b; Strong) Functional impairment (Level 2b; Strong) ED unresponsive to pharmacotherapy (Level 2a; Strong) Manual modelling (Wilson's manoeuvre) if curvature >30°. If after manoeuvre curvature >30° persists proceed with tunical plication/PIG/PEG (Strong) Do not use ‘sliding’ technique due to the significant risk of life changing complications (i.e., glans necrosis) (Strong)
Preferred prosthesis	IPP is preferred (Expert opinion)	IPP associated with higher satisfaction and lower persistent curvature compared with malleable Similar outcomes with commercially available IPP models	Inflatable prosthesis is preferred (Level 3; Grade C)	IPP classically considered more effective Malleable with similar satisfaction rates Patient and surgeon preference should determine device type and model No real difference between the available IPPs

IPP, inflatable penile prosthesis; PEG; plaque excision and grafting; PIG, plaque incision, and grafting.

* Adjunctive procedures may include manual modelling, plication, incision, or grafting.

Both authors applied the Appraisal of Guidelines for Research and Evaluation II (AGREE II) tool independently to assess guideline quality.

## Results

### Definition and Pathophysiology

All four guidelines describe PD as an acquired fibrotic disorder of the penis, typically associated with penile pain, curvature or deformity, ED, and psychological distress. A consistent aetiological narrative is offered across documents, characterising PD as a pathological wound‐healing response to repeated microtrauma, resulting in localised fibrosis of the tunica albuginea [1, 25, 26, 27]. All guidelines also acknowledge a genetic component, with the 2025 EAU update notably identifying specific genes implicated in PD pathogenesis [26]. Genetic studies in PD have implicated dysregulation of extracellular matrix remodelling (*MMP2, MMP9, COL1A1*), cytoskeletal and adhesion pathways (*ACTA2, DES, ITGB1, CTTN, FLN, RHOA, ARHGDIA*), profibrotic signalling (*TGFB1, SMAD7*), and wound‐healing/immune mediators (*MCP1, RANTES, AP1*), underscoring a multifactorial genetic basis [30].

Several associated comorbidities are highlighted, including Dupuytren's contracture, diabetes mellitus, hypertension, dyslipidaemia, ischaemic cardiomyopathy, hypogonadism, ED, prior pelvic surgery, and tobacco and alcohol use [25, 26]. In addition to physical symptoms, all panels recognise the negative impact of PD on psychological well‐being and quality of life, with depression and interpersonal difficulties frequently reported.

### Natural History and Disease Phases

There is consensus across all guidelines in describing the biphasic course of PD, comprising an initial ‘active’ phase followed by a ‘stable’ phase [31]. The active phase typically features penile pain, palpable induration, evolving deformity, and variable onset of ED. Curvature progression occurs in ~50% of patients during this phase, while spontaneous improvement is uncommon, reported in less than 10–15% of cases. Pain, in contrast, tends to resolve in most patients.

While all guidelines agree that surgical correction should be considered only after disease stabilisation, there is variation in how stability is defined. All panels concur that deformity should remain unchanged for at least 3–6 months before surgery. However, the minimum time from symptom onset used to define stable disease varies: the AUA suggests >12 months, whereas CUA and ISSM recommend >6–12 months and EAU >9–12 months [1, 25, 26, 27].

### Diagnostic Evaluation

All guidelines emphasise that evaluation and treatment of PD should be performed by clinicians experienced in its management, particularly when surgical intervention is being considered. The CUA guidelines underscores the importance of referral to subspecialised physicians for surgical management, and the ISSM further recommends that complex procedures, such as grafting, be undertaken by surgeons with specific expertise.

Due to the limited high‐quality evidence, diagnostic recommendations are largely based on expert consensus. A detailed medical and sexual history, including an assessment of deformity, erectile function, and symptom bother, is universally advised. All panels endorse a thorough physical examination of the genitalia as mandatory for diagnosis. The use of validated instruments such as the International Index of Erectile Function (IIEF) [32] and the Peyronie's Disease Questionnaire (PDQ) [33] is discussed, although not universally mandated. Notably, the ISSM guidelines explicitly recommends use of the PDQ to establish baseline symptomatology and monitor outcomes [25].

There is uniform agreement that when invasive treatment is planned, objective assessment of curvature is essential, including curvature, presence of indentations and length loss. In‐office intracavernosal injection (ICI) with goniometric measurement is regarded as the ‘gold standard’ by all panels. The CUA and earlier versions of the EAU guidelines allowed for patient‐provided photographs with protractor‐based angle estimation as an alternative, although this method is considered less accurate [27, 34, 35]. The updated EAU guidelines strongly favours in‐office ICI evaluation due to its superior accuracy, despite being supported only by low‐level evidence [26]. The AUA and ISSM also recommend ICI, while acknowledging that home photography may be sufficient in select cases [1, 25].

The role of penile ultrasonography, with or without Doppler (penile duplex Doppler ultrasonography [PDDU]), differs across guidelines. The EAU recommends its use only in the context of ED evaluation, explicitly advising against its use for plaque characterisation due to a lack of evidence for clinical utility [26]. Conversely, the AUA, ISSM and CUA consider PDDU optional for assessing plaque morphology and haemodynamic parameters [1, 25, 27]. Other imaging modalities such as MRI, CT, and plain radiography are discouraged by the EAU, ISSM and CUA due to limited clinical benefit and unnecessary cost or radiation exposure [25, 26, 27].

Penile biothesiometry is briefly addressed in the AUA and ISSM guidelines as a potential adjunct in patients reporting sensory deficits. While not routinely indicated, it may be useful for establishing a baseline assessment of penile sensitivity in select cases [1, 25]. This modality is not discussed in the EAU and CUA guidelines [26, 27].

### Psychological Assessment

All Guidelines panels recognise the psychological impact of PD, with depression, anxiety, and reduced self‐esteem commonly reported [6]. The use of instruments such as the PDQ, which includes a domain assessing psychological bother, is highlighted as a way to assess the impact on mental health. Although none of the guidelines issue formal statements mandating mental health screening or referral, all suggest that referral to a mental health professional should be considered when significant distress is identified [1, 25, 26, 27].

### Treatment

All Guidelines panels explicitly iterate the importance of shared decision‐making as it pertains to treatment selection for PD. The treating physician must inform patients on potential risks, benefits, and treatment alternatives during the initial consultation and subsequent interactions. No single treatment modality is considered the standard of care and given the benign nature observation alone is an appropriate treatment decision when made by the adequately informed patient. Treatment algorithms are included within the AUA, CUA, and EAU guidelines to assist clinicians with diagnosis and treatment options [1, 25, 26, 27].

### Non‐Surgical Treatment

#### Oral Therapy

Active treatment approaches are differentiated based on levels of invasiveness. There is consensus among guidelines panels that oral therapies lack supportive evidence [1, 25, 26, 36]. The AUA, CUA and EAU panels recommend NSAIDs as first‐line therapy for pain in the acute phase, which is the defining feature for many patients [1, 26, 27]. The CUA and EAU panel also discusses the potential role for phosphodiesterase type 5 inhibitors (PDE5i) such as tadalafil to optimise concurrent ED, which is common in men with PD, noting there is also limited animal and human data regarding potential benefits with respect to fibrosis [26, 27]. The AUA, CUA and EAU Guidelines panels recommend against oral therapy with vitamin E (+/− L‐carnitine), tamoxifen, procarbazine, and omega‐3 fatty acids due to a clear lack of efficacy [1, 26, 27]. The EAU panel recommends against potassium para‐aminobenzoate (POTABA), pentoxifylline, and colchicine [26], whereas the AUA and CUA Guidelines panels consider these agents as ‘potential options’, along with co‐enzyme Q10, when used as monotherapy or in combination with other treatment modalities (while still acknowledging the clear lack of sufficient evidence with respect to treatment efficacy) [1]. The ISSM guidelines do not recommend any oral therapy as primary treatment due to the lack of proven benefit in rigorous clinical studies to date [25].

#### Topical Therapy

All Guidelines panels point to the lack of sufficient evidence to support topical therapies such as verapamil or H‐100 (nicardipine, superoxide dismutase, and emu oil) [1, 25, 26, 27]. Electromotive drug therapy (iontophoresis) is not recommended by the AUA, CUA and EAU Guidelines panels based on lack of sufficient evidence and burden of administration [1, 26, 27]. Using a slightly different take, the ISSM Guidelines panel underscores the lack of proven benefit and suggests that the decrease in curvature seen in some series may be due to the energy delivered to the tissue [25].

#### Extracorporeal Shockwave Therapy (ESWT) and Radiation Therapy

The mechanism by which ESWT exerts its effect in PD remains unclear. Two main hypotheses have been proposed. The first suggests that ESWT directly damages and remodels the penile plaque. The second proposes that the therapy increases local circulation through heat generation, triggering an inflammatory response and enhanced macrophage activity, ultimately leading to plaque lysis and resorption [34]. There is general agreement that ESWT may be offered for pain, but not curvature correction, even though the treatment protocol remains unclear [1, 25, 26, 27]. The AUA emphasises the low utility given that the natural progression of PD results in pain resolution for most patients. Radiation is not recommended by the AUA and CUA guidelines given potential associated risks which outweigh any proven benefits [1, 27]. This is not addressed by the ISSM or EAU guidelines [25, 26].

#### Penile Traction Therapy (PTT) and Vacuum Erection Device (VED)

According to the EAU guideline, PTT or VED may be employed either as monotherapy or in combination with other treatments to reduce the penile deformity [26]. This recommendation is supported by several small studies reporting improvements in both curvature and penile length, with only mild side effects. Nonetheless, the strength of the recommendation is graded as ‘weak’, reflecting the limited robustness of the available evidence. The CUA guidelines also supports the use of PTT but underscores the absence of standardised treatment protocols, particularly regarding device type and duration of application [27]. The AUA Guidelines panel suggests that mechanical therapies including PTT and VED are ‘possibly promising’, but further replication of the available study data is required [1]. This is in line with recommendations by the ISSM Guidelines group as well [25]. In addition to current ISSM guidelines, in 2021 the ESSM published position statements on the use of PTT for PD. Specifically, PTT was considered a safe and well‐tolerated non‐surgical treatment for PD, with reported curvature improvements ranging from 4° to 31.2° (~13%–41%). While PTT may offer benefits following surgical correction of PD, further robust studies are required to define its efficacy and optimal indications. However, current evidence remains limited, heterogeneous, and insufficient to support routine clinical use, either as a primary therapy or as an adjunct to oral or intralesional treatments, underscoring the need for continued investigation into its biological mechanisms and therapeutic role [37].

#### Intralesional Injections

Intralesional injection therapy has been extensively studied for the management of PD, with multiple pharmacological agents explored over recent decades, including hyaluronic acid, botulinum neurotoxin‐A (BoNT‐A), corticosteroids, verapamil, interferon alpha 2b (IFN‐α2b), and collagenase clostridium histolyticum (CCH). The latter is the only intralesional therapy to have received national regulatory approval in the United States and Europe, although it is currently unavailable in Canada and the European Union [38].

### Corticosteroids, Hyaluronic Acid, and BoNT‐A

There is strong consensus across all guidelines regarding the lack of efficacy for corticosteroids, hyaluronic acid, and BoNT‐A. EAU guidelines explicitly recommend against (Strong, Level 3c) the use of intralesional corticosteroids [26], while the AUA, CUA and ISSM guidelines refrain from firm recommendations but cite significant limitations in the available data [1, 25, 27]. Similarly, the AUA guidelines highlight insufficient evidence to support hyaluronic acid or BoNT‐A therapy in PD management [1]. However, while not supporting BoNT‐A therapy, the EAU guidelines state that intralesional hyaluronic acid may be used to improve pain, penile curvature and IIEF scores (level 2b), and that a combination of oral and intralesional hyaluronic acid treatment improves penile curvature and plaque size (level 1b), but only within the settings of a clinical trial (Strong) [26].

### Intralesional Verapamil

Intralesional verapamil, a calcium‐channel blocker, is proposed to modulate extracellular matrix remodelling and reduce fibrosis. The AUA guidelines states that verapamil may be offered to patients but emphasises the inconsistent and low‐quality evidence supporting its use, with an unclear balance of benefits and risks [1]. Adverse effects are generally mild and transient, and some studies have reported improvements in penile pain, although such observations often lack control group comparisons. The ISSM guidelines adopt a similar cautious stance [25]. The CUA guidelines classifies verapamil, together with IFN‐α2b, as a second‐line intralesional therapy, and highlights that variations in injection protocols (including volume, frequency, concentration, and treatment duration) are likely to affect outcomes [27]. In contrast, the EAU guidelines do not support the clinical use of intralesional calcium channel blockers (verapamil or nicardipine) due to the lack of robust evidence of curvature improvement [26].

### Intralesional IFN‐α2b

Interferon alpha 2b is another intralesional agent that may inhibit fibroblast proliferation and modulate collagen deposition [39]. All four guidelines support IFN‐α2b as a treatment option for stable‐phase PD [1, 25, 26, 27], despite citing mild adverse effects, which include sinusitis and flu‐like symptoms, which can be effectively treated with NSAIDs before IFN‐α2b injection. Notably, although the CUA Guidelines panel lists IFN‐α2b as a second‐line intralesional therapy, its use in Canada is uncommon due to excessive cost and potential adverse effects, including sinusitis and flu‐like symptoms [27]. This recommendation is based on modest benefits demonstrated in clinical trials, particularly the multicentre, randomised, placebo‐controlled study by Hellstrom et al. [40] in 2006.

The AUA and CUA guidelines specify that suitable candidates for IFN‐α2b therapy should have non‐calcified plaques and penile curvature >30° [1, 27]. Similarly, the EAU guidelines suggest offering intralesional therapy with IFN‐α2b to patients with stable curvature dorsal or lateral >30° seeking a minimal invasive procedure [26]. The AUA further notes that clinicians should manage expectations regarding the degree of curvature improvement, citing an average reduction of ~13.5° compared with a 9° reduction in placebo groups [1]. Other reported benefits include potential improvements in penile pain and vascular haemodynamics, which are consistently emphasised across guidelines [1, 25, 26].

### Intralesional CCH

Among intralesional treatments, CCH remains the most strongly supported therapy across all guidelines, owing to robust data from two pivotal double‐blind, randomised, placebo‐controlled trials published in 2013 [41]. Subsequent clinical studies have reinforced these findings, demonstrating significant curvature reduction and improvements in penile deformity [11]. The AUA guidelines recommends CCH for patients with stable‐phase PD, penile curvatures between 30 and 90°, and good erectile function [1]. The EAU similarly endorses CCH use for patients seeking non‐surgical treatment but specifies that the curvature should be dorsal or lateral rather than ventral. Likewise, the EAU guidelines specify CCH is no longer available in the European Union [26]. The CUA and ISSM guidelines identify hourglass deformities with hinge effects and significant plaque calcification as exclusion criteria for CCH therapy [25, 27]. Notably, evidence remains insufficient regarding CCH use in the acute phase of PD, ventral curvature, or isolated hourglass deformities, and none of the guidelines overtly recommend against its use in these contexts; rather, they highlight the lack of robust data to guide practice. All panels emphasise the importance of patient counselling prior to CCH treatment, outlining potential side effects such as penile bruising, swelling, haematoma, pain, and the risk of penile fracture, the latter being explicitly noted in AUA Guidelines Statement number 9 [1].

### Platelet‐Rich Plasma (PRP)

Platelet‐rich plasma has been investigated in only a few small human studies assessing its effect on penile curvature, plaque size, PDQ, and IIEF, with inconclusive results. Its role in PD remains unproven, and current evidence supports considering PRP as experimental [26]. Preliminary data from an ongoing phase 2b randomised placebo‐controlled crossover trial (25 patients enrolled, target 80) showed no significant difference in curvature at 3 months between nine men treated with PRP and eight receiving placebo [42]. At present, the AUA, ISSM, and CUA guidelines provide no recommendations on the use of PRP in PD [1, 25, 27], while the EAU guideline states that intralesional PRP or hyaluronic acid, alone or combined with oral therapy, should not be used outside clinical trials to reduce curvature, plaque size, or pain [26].

### Surgical Management

Surgical treatment for PD encompasses penile plication, plaque incision or partial excision with grafting, and penile prosthesis implantation, with adjunctive straightening manoeuvres where indicated. Surgery is considered in patients who either fail conservative management, have significant deformity precluding intercourse, or express a strong preference for definitive correction. The primary objective of surgical intervention is to restore penile straightness and function, rather than to alleviate pain.

All Guidelines panels agree that surgery should be offered only after the disease has stabilised. Although there is consensus that penile curvature should remain stable for at least 3 months, the ISSM recommends waiting at least 6 months, while the AUA suggests waiting at least 12 months from symptom onset, with 3–6 months of stability before surgery [1, 25]. The 2022 ESSM Position Statement on Surgical Treatment of PD also recommends that surgery should only be performed in patients with stable disease for at least 6–12 months to minimise the risk of further disease progression or recurrence [43]. This variation underscores persistent heterogeneity in clinical practice and highlights the need for standardised definitions of disease stability to improve consistency in outcome reporting across studies.

#### Surgical Algorithms and Procedure Selection

Early surgical algorithms, notably from Levine and Lenting [44], proposed stratifying treatment decisions based on curvature severity, presence of hourglass deformity or hinge effect, and baseline erectile function. Subsequent research has supported this objective approach to enhance postoperative outcomes [45, 46, 47].

Guidelines vary in specific recommendations regarding the choice between plication vs grafting techniques. The CUA, EAU and ISSM suggest reserving plaque incision with grafting for men with more severe deformity, while plication may be appropriate for those with milder curvature [25, 26, 27]. The ESSM similarly states that grafting procedures should be considered in cases with curvature >60°, ossified plaques, significant hourglass deformity, or when plication would result in a loss of >20% of penile length [43]. Terminology used to define curvature suitable for plication varies, including descriptors such as ‘non‐severe’, ‘reasonably correctable’, or ‘minimal to moderate’. The AUA does not offer specific recommendations regarding the decision to pursue plication vs grafting [1].

The ESSM emphasises the importance of individualised preoperative counselling, underlining that patients must be informed that surgical treatments often cannot restore the penis to its pre‐disease dimensions and that penile shortening is reported by >70% of patients prior to surgery. Furthermore, thorough discussion of potential complications—including ED, sensory changes, and residual curvature—is crucial for setting realistic expectations and improving postoperative satisfaction [43].

No formal guidelines strongly favours one surgical technique over another for either plication or plaque incision and grafting, reflecting the absence of high‐quality comparative data. The EAU specifies that a degloving incision is considered standard for surgical exposure, although alternative approaches, such as ventral incisions, have been described. Additionally, the EAU emphasises the use of non‐absorbable or slowly absorbable sutures to reduce the risk of curvature recurrence in plication procedures [26]. Regarding grafting, none of the societies endorses a single preferred graft material. The CUA, EAU and ISSM strongly recommend against the use of synthetic grafts, such as Dacron™ and Gore‐Tex™, due to the association with fibrosis and infection [25, 26, 27]. The ESSM explicitly reinforces this recommendation, advising against synthetic materials because of their higher rates of infection, fibrosis, contracture, and inflammatory reactions [43]. Various graft options remain under investigation. The ESSM highlights promising results with self‐adhesive collagen fleece (e.g., TachoSil™), which reduces operative time and eliminates the need for suturing, achieving penile straightening rates of up to 83%. However, long‐term outcomes remain unproven. Small intestinal submucosa remains one of the most commonly used xenografts, while autologous options like saphenous vein and buccal mucosa grafts have shown favourable results but require longer operative time and additional surgical sites [43]. The ESSM also notes that no single tunical incision type (e.g., transverse, H‐incision, or double‐Y incision) has proven superior and emphasises that larger defects increase the risk of postoperative ED [43].

#### Penile Prosthesis and Straightening Techniques

There is general agreement among the panels that an inflatable penile prosthesis (IPP) is the preferred device for men with combined PD and medication‐refractory ED [25, 26, 27, 43], although the EAU notes that satisfaction rates are comparable between inflatable and malleable prostheses [26] and the AUA guidelines do not indicate a preference toward either malleable penile prostheses or IPPs [1]. Prosthesis implantation serves both to restore erectile rigidity and to facilitate penile straightening via adjunctive techniques.

A widely accepted surgical algorithm for combined PD and ED management was proposed by Levine and Dimitriou [48] in 2000. The ISSM and EAU guidelines reference similar treatment pathways [25, 26]. For residual curvature >30° after IPP, manual modelling is recommended as the initial corrective step. If significant curvature persists, options include penile plication or plaque incision with or without grafting. According to ISSM guidelines, if a tunical defect >2 cm results from plaque incision, grafting over the defect is advised [25, 49].

The ESSM emphasises that IPP offer superior outcomes in terms of curvature correction, girth restoration, and concealability compared to semi‐rigid implants, making them the preferred choice in PD surgery. Furthermore, ESSM confirms that surgical correction after prior CCH injections is feasible without significantly increasing postoperative complication rates, although surgery may be more technically challenging if performed within 3 months of the last CCH injection [43].

‘Length‐restoration’ procedures, such as the sliding technique [50], are discussed cautiously. The ESSM underscores that such techniques should be used cautiously and only in specialised centres due to increased risks of glans ischaemia or necrosis [43]. Instead, the EAU guidelines recommend against the ‘sliding’ technique due to reported cases of glans necrosis because of the concomitant mobilisation of the neurovascular bundle and urethra [26]. However, the guidelines recognises that new approaches such as the Modified Sliding Technique (MoST) [51], Multiple‐Slit Technique (MUST) [52] or Multiple‐Incision Technique (MIT) [53] techniques, can be used, but only by experienced high‐volume surgeons and after full patient counselling [26].

#### Surgical Risks and Patient Counselling

All Guidelines panels emphasise the need for thorough preoperative counselling, addressing potential surgical risks such as penile shortening, ED, altered penile sensation, and persistent or recurrent curvature [1, 25, 26, 27]. The EAU, CUA and ISSM specifically discuss the concept of a ‘functionally straight’ penis, defined as curvature within 20° of straightness, rather than achieving a perfectly straight penile shaft, as first proposed by Levine et al. [25, 26, 27, 54]. ED and sensory loss are less common following plication, particularly when dissection of the neurovascular bundle is avoided. The ESSM highlights that glans hypoaesthesia is a frequent complication, reported in up to 53% of cases, although it is often transient and resolves within 1 year. Palpable sutures and graft aneurysm can also occur, affecting patient satisfaction. Bulge or indentation deformities are reported in 9–16.7% of patients. Glans ischaemia or necrosis, although rare, is a severe complication following surgery and mainly associated with lengthening procedures [43].

Risks associated with IPP include device infection, mechanical malfunction, erosion, and urethral perforation, especially if manual modelling is performed [1, 25, 26, 27].

## Discussion

Peyronie's disease remains a challenging condition in both diagnosis and management. Despite longstanding recognition, high‐quality evidence guiding treatment is scarce. Most studies are small, methodologically limited, and lack standardised outcomes, leading to reliance on expert consensus rather than robust clinical data [20, 21, 22, 43]. This is compounded by incomplete understanding of the fibrotic mechanisms underlying PD.

Our review of current guidelines and the ESSM Position Statement highlights broad consensus on diagnostic principles. Thorough history and physical examination are essential, while ICI with objective curvature assessment remains the ‘gold standard’ before invasive therapy [1, 25, 26, 27, 43]. However, patient recollection or photographic methods yield variable accuracy [20, 35, 43], and no universally accepted measurement protocol exists. Given that most treatments provide only 10–20° curvature improvement [20]. Reproducible assessment methods are crucial. Novel tools such as three‐dimensional photography and smartphone‐based applications may enhance diagnostic precision in the era of telemedicine [43, 55, 56].

The psychological burden of PD is substantial for both patients and partners. Instruments such as the PDQ help quantify distress and monitor treatment response [1, 9, 25, 26, 27, 33], yet they are limited for individuals outside heterosexual partnerships or not engaging in penetrative sex [20]. Broader validated instruments inclusive of diverse populations and partner perspectives remain needed [2, 3, 43, 57].

Variation in clinician expertise, access to treatments, and familiarity with PD care contributes to inconsistent management [23, 24]. To address this, the AUA, ISSM, CUA, and EAU have developed guidelines to standardise care, though most recommendations still rely on expert opinion [1, 25, 26, 27].

Despite broad agreement on disease biology and overarching principles, differences across guidelines can meaningfully influence patient management. In particular, North American guidelines (AUA, CUA) adopt a more permissive stance toward selected oral and intralesional therapies despite limited supporting evidence, whereas the EAU and ISSM discourage most conservative treatments outside clinical trials. Consequently, patients with similar disease characteristics may receive different treatments depending on which guideline informs care. These discrepancies are further amplified by regional treatment availability, most notably the absence of collagenase in the European Union, and by variation in how disease stability is defined, which may affect the timing of surgical referral. Importantly, these differences arise not from disagreement on PD pathophysiology, but from heterogeneity in data acquisition, study quality, and outcome definitions, as well as healthcare system constraints and regulatory factors. Addressing this variability will require harmonisation of diagnostic standards, adoption of core outcome measures that incorporate functional and psychological endpoints, and collaborative multicentre registries or pragmatic trials stratified by disease phase and deformity phenotype, rather than further proliferation of guideline documents. Furthermore, given the scarcity of high‐quality prospective randomised trials, the grading of recommendations in PD guidelines often relies on expert opinion. This can lead to variability across different guidelines.

There is strong agreement that oral agents such as vitamin E and antioxidants lack efficacy and should not be used [1, 25, 26, 27, 43]. PDE5i may improve erectile function but not disease progression. ESWT can alleviate pain during the acute phase but has no proven effect on curvature or plaque size and lacks standardised treatment protocols.

Penile traction therapy may offer modest improvements in curvature and length [26, 37], but data remain limited and protocols inconsistent; it should not be used as monotherapy until stronger evidence emerges. Among intralesional therapies, CCH shows the strongest evidence, producing modest but significant curvature reductions [11], although access remains limited outside the United States [38]. Hyaluronic acid has recently been approved for acute‐phase PD in Italy, showing a favourable safety profile and potential functional benefits [58, 59, 60, 61].

Surgery remains the definitive option for men with stable PD and significant deformity or functional limitation. The ESSM emphasises preoperative counselling, as many patients already perceive penile shortening preoperatively [43]. Plication techniques are suited for milder curvature, whereas plaque incision and grafting are preferred for severe or complex deformities. However, no guideline specifies the optimal approach for hourglass deformity in men undergoing IPP, an important gap in practice. Synthetic grafts are discouraged due to fibrosis and infection risks, while newer self‐adhesive collagen fleece grafts show promise but lack long‐term outcomes.

An IPP is the treatment of choice for PD with refractory ED [1, 25, 26, 27, 43]. Adjunctive manoeuvres such as modelling, tunical incision, or grafting can correct residual curvature >30° [43, 49] and home modelling may suffice for deformities <45° [62]. Surgery remains feasible after prior collagenase injections, though technically demanding.

Future advances will likely stem from elucidating PD's fibrotic mechanisms and re‐purposing systemic antifibrotic agents under study for pulmonary fibrosis [63]. The Fifth International Consultation on Sexual Medicine (ICSM, 2024) is expected to update consensus statements on diagnostic standards, medical therapies, and surgical techniques [64]. Meanwhile, improving public awareness and reducing stigma are critical for earlier diagnosis. A multidisciplinary approach—integrating urologists, sexual medicine experts, psychologists, and partners—remains essential to optimise outcomes.

## Conclusions

Four major international guidelines standardise the evaluation and management of PD. They agree that optimal care relies on specialist expertise, detailed clinical assessment, and objective curvature measurement before invasive treatment. Oral medications lack efficacy and are not recommended, whereas intralesional injections represent valuable non‐surgical options for selected patients. Surgery should be reserved for stable disease, with IPP as the preferred solution in men with refractory ED. Continued translational and clinical research is vital to refine therapeutic strategies and enhance patient selection.

## Disclosure of Interests

None.

## Funding

None.

## Supporting information


**Data S1** Details on the development processes of the major international guidelines on PD.
