# Supplementary material for: Guideline of guidelines: Peyronie's disease
Source: BJU Int. 2026 Mar 8;137(5):770–82. doi: 10.1111/bju.70201 (PMC13071548; doi:10.1111/bju.70201)
Supplement: Supplementary file 1 — Data S1 Details on the development processes of the major international guidelines on PD. [file BJU-137-770-s001.docx]

**Supplementary Material: details on the development processes of the major international guidelines on Peyronie’s disease**

The **AUA guideline**, published in 2015, aimed to assist clinicians in recognizing PD, conducting appropriate diagnostic evaluations, and providing individualized treatment to optimize symptom control, sexual function, and quality of life for patients and their partners(1). The panel of 14 experts in sexual medicine and evidence synthesis conducted a systematic review of the PubMed, Embase, and Cochrane databases spanning 1965 to 2015. Of the screened articles, 281 met inclusion criteria and informed the development of 22 guideline statements—three focused on diagnosis and 19 on treatment. Recommendations were graded A to C based on evidence strength, supplemented by consensus statements when data were lacking.

In the same year, the ISSM convened the Fourth International Consultation on Sexual Medicine (ICSM) in Madrid (2). A panel of 10 experts reviewed literature up to 2015, including existing AUA and EAU guidelines. Selected articles were used to revise and expand upon the recommendations established during the prior ICSM in 2009. The consultation yielded seven summary statements, supported by additional diagnostic and therapeutic recommendations elaborated within the guideline text. Levels of evidence and expert consensus were assigned to each recommendation.

The Canadian Urological Association (CUA) guideline on Peyronie’s disease and congenital penile curvature was published in 2018 (3). Developed by a panel of six sexual medicine experts, its aim was to provide practical recommendations for managing PD within the Canadian healthcare system. The panel conducted a systematic review of the literature up to 2017, excluding pre-clinical studies. Evidence quality was assessed using the International Consultation for Urologic Disease (ICUD)/World Health Organization (WHO)–modified Oxford Centre for Evidence-Based Medicine grading system (levels 1–4), with final recommendations assigned consensus grades ranging from A to D (D = consensus statement or no recommendation possible), following an approach similar to the AUA guideline (1). Given the limited quality of available data, grade A and B recommendations were infrequent. The guideline provides detailed recommendations within its discussion and also includes a treatment algorithm.

In 2025, the EAU published updated guidance on sexual and reproductive health, including a dedicated section on penile curvature and PD(4). This document updates prior EAU guidelines from 2020 to 2024. A panel of 17 experts conducted an updated systematic review of new research published in 2021–2024 in Medline, EMBASE, and Cochrane Libraries. The guidelines’ recommendations focused on key clinical decisions that would impact patient care most. Each recommendation’s strength was evaluated based on three factors: the trade-offs between benefits and drawbacks of different treatment approaches, the quality and reliability of the available evidence, and the diverse preferences and values of patients. Levels of evidence were rated on a 1 to 5 scale, and guideline statements were categorized as “strong” or “weak,” with detailed rationale provided in the accompanying text.

BIBLIOGRAPHY

1. Nehra A, Alterowitz R, Culkin DJ, Faraday MM, Hakim LS, Heidelbaugh JJ, et al. Peyronie’s Disease: AUA Guideline. J Urol [Internet]. 2015 Sep 1 [cited 2025 Jun 20];194(3):745. Available from: https://pmc.ncbi.nlm.nih.gov/articles/PMC5027990/

2. Chung E, Ralph D, Kagioglu A, Garaffa G, Shamsodini A, Bivalacqua T, et al. Evidence-based management guidelines on peyronie’s disease. Journal of Sexual Medicine [Internet]. 2016 Jun 1 [cited 2025 Jun 20];13(6):905–23. Available from: https://pubmed.ncbi.nlm.nih.gov/27215686/

3. Bella AJ, Lee JC, Grober ED, Carrier S, Benard F, Brock GB. 2018 Canadian Urological Association guideline for Peyronie’s disease and congenital penile curvature. Canadian Urological Association Journal [Internet]. 2018 May 1 [cited 2025 Sep 7];12(5):E197. Available from: https://pmc.ncbi.nlm.nih.gov/articles/PMC5966931/

4. Salonia A, Capogrosso P, Boeri L, Cocci A, Corona G, Dinkelman-Smit M, et al. European Association of Urology Guidelines on Male Sexual and Reproductive Health: 2025 Update on Male Hypogonadism, Erectile Dysfunction, Premature Ejaculation, and Peyronie’s Disease. Eur Urol [Internet]. 2025 Jul 1 [cited 2025 Jun 20];88(1):76–102. Available from: https://linkinghub.elsevier.com/retrieve/pii/S0302283825002118
